# 

**Published:** 1845-10

**Authors:** 


### CONTENTS

OF THE

### MEDICAL EXAMINER.

NEW SERIES—NO. X.—OCTOBER, 1845.

### ORIGINAL COMMUNICATIONS.

A series of cases illustrative of some new views upon the Pathology and Treatment of Epidemic Dysentery. By O. Bailey, M. D., of Lancaster Co., Pennsylvania, - - - - - - - 583

A case of Anasarca and Ascites, depending on the pressure of a Tumour upon the large blood vessels. Reported by Laurence Turnbull, M.D. Resident Physician of Blockley Hospital, Philadelphia, - 592

### CLINICAL LECTURES AND REPORTS.

### PHILADELPHIA HOSPITAL,

*Saturday, February* 22, 1845.

Last clinic of the course of Professor Dunglison.

Ascites, - - - - - - - - - - - - - - 595

Endocarditis, complicated with valvular disease, - - - - 596

### BIBLIOGRAPHICAL NOTICES.

Lectures on the Principles and Practice of Physic; delivered at Kings College, London. By Thomas Watson, M. D. &c. &c. Second American from the Second London Edition, Revised, with Additions, by D. Francis Condie, M. D., Secretary of the College of Physicians; author of a Treatise on Diseases of Children, etc. etc. 602

An Elementary Treatise on Midwifery: or Principles of Tokology and Embryology. By Alf. A. L. M. Velpeau, M. D., &c. &c. Translated from the French by Charles D. Meigs, M. D., member of the American Philosophical Society; Professor of Midwifery in the Jefferson Medical College, &c. &c. Third American edition, with notes and additions by William Harris, M. D., Member of the American Philosophical Society; Lecturer on Midwifery and the Diseases of Women and Children, &c. &c. - - 607

Modern Cookery, in all its branches, reduced to a system of Easy Practice for the use of private families; in a series of receipts, which have been strictly tested, and are given with the most minute exactness. By Eliza Acton. Illustrated with numerous wood cuts. To which are added directions for carving, garnishing, and setting out the table; with a table of weights and measures; the whole revised and prepared for American Housekeepers, by Mrs. S. J. Hale. From the second London edition, - - - - - 611

Anecdota Sydenhamiana: Medical notes and observations of Thomas Sydenham, M. D., hitherto unpublished, - - - - 613

Manual of Clinical Medicine: Special Pathology and Therapeutics, regarded in a Clinical point of view. By Dr. Charles Canstatt, &c. &c. Second augmented edition, - - - - - - - - 615

The Anatomy and Diseases of the Breast. With numerous plates. By Sir Astley Cooper, Bart., F. R. S., Sergeant Surgeon to his Majesty; Consulting Surgeon of Guy's Hospital; Lecturer on Anatomy and Surgery, &c. &c. To which are added his various Surgical papers, now first pnblished in a collected form, - - 616

Half Yearly Abstract of the Medical Sciences, &c. &c. Edited by Wm. H. Ranking, M. D., Cantab. Vol. 1. January—June, 1845, - 617

### EDITORIAL.

The Works of Hippocrates and Galen, - - - - - - - - 618

New Orleans Medical and Surgical Journal, devoted to Medicine and the Collateral Sciences, - - - - - - - - - - - - 619

New York Medical Intelligencer, - - - - - - - - - 619

Annual circulars of Medical Colleges.

1.—School of Medicine and Surgery of Montreal (Canada.) - - 620

2.—Annual Announcement of Jefferson Medical College, - - - 620

3.—Catalogue and Circular of the Albany Medical College, - - 620

4.—Annual Circular of the Washington University of Baltimore, Session, 1845–6, - - - - - - - - - - - - 621

5.—Report on the Medical Department of the University of Pennsylvania, for the year 1845, - - - - - - - - - - 621

6.—Third Annual Announcement and Catalogue of the Rush Medical College, Chicago, Ill. - - - - - - - - - - 622

Dr. Greenhill's Prospectus, - - - - - - - - - - 622

Death of Professor Richardson, of Transylvania University. - - - 623

### RECORD OF MEDICAL SCIENCE.

Post-Mortem Caloricity of Yellow Fever. By Bennett Dowler, M. D. of New Orleans, - - - - - - - - - - - - 624

Adulteration of Veratrine with Lime, - - - - - - - - 626

On Intra-thoracic Solid Tumours, - - - - - - - - - 626

Illustrations of some of the forms of Sudden Death. By D. J. T. Francis, M. B.

1.—Cases in which the Cause of Death was seated in the Brain, - 630

2. Cases in which there was congestion of the right heart and lungs, 632

3. Cases in which death was the result of syncope, - - - 634

4. Cases in which death was the result of epilepsy, - - - 635

The Nature and Development of Accidental Productions. By Ch. Baron, M. D. - - - - - - - - - - - - 636
A Memoir on Uretro-Plastie, - - - - - - - - - 640
Death from Aconite—inquest, - - - - - - - - - 641
Punctured wound of the Abdomen, - - - - - - - - 643
On the Modification which the Blood undergoes during inflammation. By MM. Robert Latour and Collignon, - - - - - - - 644
Peritonitis following examination for uterine polypus, - - - - - 644
Trial of Goule for murder, - - - - - - - - - - - 644
Death of Professor Graham, of Edinburg, - - - - - - - 646
Death of Dr. John Houston, - - - - - - - - - - - 646

